# Astrocytes generated from patient induced pluripotent stem cells recapitulate features of Huntington’s disease patient cells

**DOI:** 10.1186/1756-6606-5-17

**Published:** 2012-05-21

**Authors:** Tarja A Juopperi, Woon Ryoung Kim, Cheng-Hsuan Chiang, Huimei Yu, Russell L Margolis, Christopher A Ross, Guo-li Ming, Hongjun Song

**Affiliations:** 1 Institute for Cell Engineering, Johns Hopkins University School of Medicine, Baltimore, MD 21205, USA; 2 Department of Neurology, Johns Hopkins University School of Medicine, Baltimore, MD 21205, USA; 3 The Solomon H. Snyder Department of Neuroscience, Johns Hopkins University School of Medicine, Baltimore, MD 21205, USA; 4 Department of Psychiatry and Behavioral Sciences, Johns Hopkins University School of Medicine, Baltimore, MD 21205, USA

**Keywords:** Huntington’s disease, Induced pluripotent stem cells, Neural differentiation, Astrocytes, Disease modeling

## Abstract

**Background:**

Huntington’s Disease (HD) is a devastating neurodegenerative disorder that clinically manifests as motor dysfunction, cognitive impairment and psychiatric symptoms. There is currently no cure for this progressive and fatal disorder. The causative mutation of this hereditary disease is a trinucleotide repeat expansion (CAG) in the Huntingtin gene that results in an expanded polyglutamine tract. Multiple mechanisms have been proposed to explain the preferential striatal and cortical degeneration that occurs with HD, including non-cell-autonomous contribution from astrocytes. Although numerous cell culture and animal models exist, there is a great need for experimental systems that can more accurately replicate the human disease. Human induced pluripotent stem cells (iPSCs) are a remarkable new tool to study neurological disorders because this cell type can be derived from patients as a renewable, genetically tractable source for unlimited cells that are difficult to acquire, such as neurons and astrocytes. The development of experimental systems based on iPSC technology could aid in the identification of molecular lesions and therapeutic treatments.

**Results:**

We derived iPSCs from a father with adult onset HD and 50 CAG repeats (F-HD-iPSC) and his daughter with juvenile HD and 109 CAG repeats (D-HD-iPSC). These disease-specific iPSC lines were characterized by standard assays to assess the quality of iPSC lines and to demonstrate their pluripotency. HD-iPSCs were capable of producing phenotypically normal, functional neurons *in vitro* and were able to survive and differentiate into neurons in the adult mouse brain *in vivo* after transplantation. Surprisingly, when HD-iPSCs were directed to differentiate into an astrocytic lineage, we observed the presence of cytoplasmic, electron clear vacuoles in astrocytes from both F-HD-iPSCs and D-HD-iPSCs, which were significantly more pronounced in D-HD-astrocytes. Remarkably, the vacuolation in diseased astrocytes was observed under basal culture conditions without additional stressors and increased over time. Importantly, similar vacuolation phenotype has also been observed in peripheral blood lymphocytes from individuals with HD. Together, these data suggest that vacuolation may be a phenotype associated with HD.

**Conclusions:**

We have generated a unique *in vitro* system to study HD pathogenesis using patient-specific iPSCs. The astrocytes derived from patient-specific iPSCs exhibit a vacuolation phenotype, a phenomenon previously documented in primary lymphocytes from HD patients. Our studies pave the way for future mechanistic investigations using human iPSCs to model HD and for high-throughput therapeutic screens.

## Background

Huntington’s disease (HD) is a fatal, progressive, neurodegenerative disorder that affects approximately 1 in 10,000 people [1]. The disease clinically manifests as motor dysfunction, typified by involuntary movements, cognitive abnormalities and psychiatric disturbances [2]. The genetic defect for this autosomal dominant disorder is a trinucleotide repeat expansion (CAG) in exon 1 of *huntingtin* (*HTT*), which results in an expanded polyglutamine tract at the N-terminal of the HTT protein [3]. Proteolytic cleaving of the abnormal HTT protein results in insoluble aggregates that form characteristic inclusions within the nucleus and cytoplasm of affected cells [4,5]. Although the wild-type HTT protein is ubiquitously expressed [6], there is a preferential accumulation of the mutant HTT protein in neurons, including medium spiny neurons of the striatum [7]. Mutant HTT aggregates have also been detected in other neural cell types such as astrocytes in the brain of HD patients [8,9]. Regional neurodegeneration characterized by striatal degeneration and cortical loss is a hallmark of HD pathology [10,11]. Other areas of the brain, such as the hippocampus, are also affected [12].

HD manifests in both a juvenile form that has a childhood onset, and a more common form that presents at the middle age. Unaffected individuals have up to 35 CAG repeats within the *HTT* gene and HD is associated with repeats of 36 or more [13]. There is an inverse correlation with CAG repeat length and onset of disease, with longer repeats (>55 CAG) associate more commonly with a juvenile onset [14]. Paternal inheritance of the *HTT* mutation may result in CAG repeat length instability and an increase in CAG repeat length [15,16]. Although HD is a defined genetic disorder and the causative mutation was identified almost two decades ago [3], the exact mechanism by which mutant *HTT* results in neuronal degeneration has yet to be determined, and major therapeutic advances have been lacking. Various *in vitro* cell culture systems [17,18] and animal models [19,20] have been developed to investigate HD pathogenesis and have provided numerous theories, such as abnormal mitochondrial bioenergetics, oxidative damage, transcriptional dysregulation and abnormal vesicle trafficking [2,5,21]. The potential role of glia cells, such as astrocytes, in the pathogenesis of HD is also being investigated [9,22-24]. For example, expression of HTT with expanded polyglutamine in astrocytes has been shown to affect glutamate transport and exacerbate neurological phenotypes in a mouse model of HD [22,23]. The cholesterol defect is also observed in astrocytes in multiple rodent models of HD [25]. A direct pathogenic role of astrocytes in the disease process of patients remains unknown.

The discovery of a combination of transcription factors that could reprogram somatic cells into cells exhibiting pluripotency has provided researchers with a revolutionary tool to study human biology and diseases [26,27]. The induced pluripotent stem cells (iPSCs) can be derived from many somatic cell types, including easily accessible dermal fibroblasts and peripheral blood lymphocytes [28,29]. Similar to human embryonic stem cells (hESCs), iPSCs can self-renew and expand indefinitely in culture [27,30]. More importantly, they share the capacity to generate any cell types in the body, a property that is particularly useful for the study of neurological diseases [31-35]. The pluripotency of iPSCs enables the production of neurons and glia from healthy individuals and from patients with diseases. This remarkable feature of iPSCs facilitates the study of brain cell types that are difficult to obtain from living individuals. Here we report the generation of iPSCs from a male patient with an adult form of HD (F-HD-iPSCs) and from his daughter with juvenile onset HD (D-HD-iPSCs). Consistent with previous reports, functional neurons can be derived from both HD-iPSCs that are phenotypically normal. However, when astrocytes were differentiated from these iPSCs, we identified a cellular vacuolation phenotype that has not been reported in neural cells, but observed in patient lymphocytes with HD. The ability of the HD-iPCSs to replicate a disease relevant phenotype that is found in primary patient tissues supports the use of patient-specific iPSCs for disease modeling and opens doors for future high-throughput screens.

## Results

### Derivation and characterization of HD-iPSC lines

**Figure 1:**
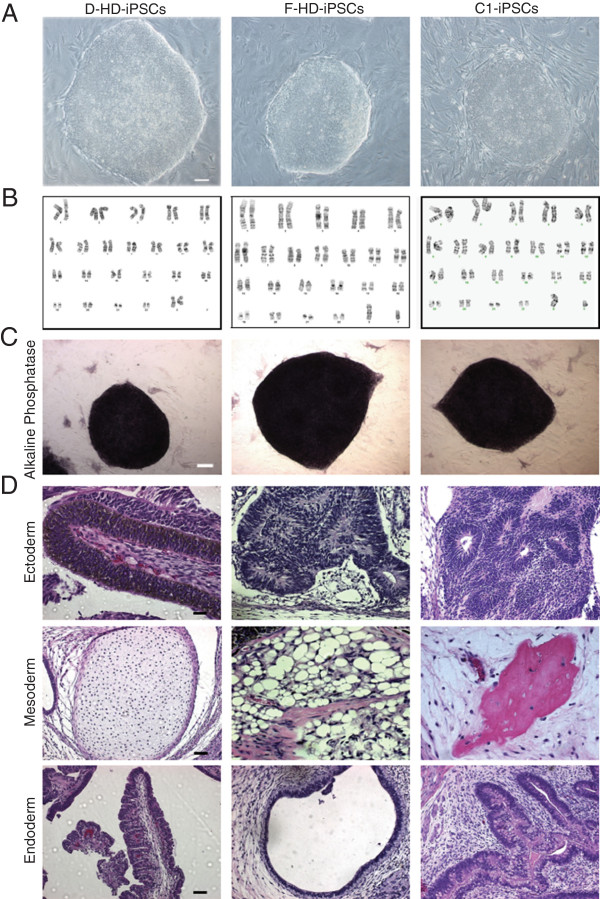
**Characterization of induced pluripotent stem cell lines (iPSCs). A, B**: iPSC lines generated from fibroblasts exhibited typical ESC morphology by phase contrast microscopy (**A**) and maintained a normal karyotype (**B**). **C**: iPSCs strongly expressed the pluripotency marker alkaline phosphatase, whereas the surrounding MEFs were negative. **D**: Hematoxylin and eosin staining of teratomas derived from iPSC lines confirmed pluripotency by the presence of tissues representing the three major germ layers: ectoderm, endoderm, mesoderm. Scale bars: 100 μm.

To derive the iPSC lines, we retrovirally introduced the four reprogramming factors (Oct3/4, Sox2, c-MYC and Klf4) [26,27] into dermal fibroblasts harvested from a male patient with adult onset HD (50 CAG repeats), his daughter with juvenile-HD (109 CAG repeats) and non-related neonatal foreskin fibroblasts (28 CAG repeats) as controls. Colonies generated from all three fibroblast cell lines exhibited typical iPSC morphology (Figure 1A), similar to conventional hESC lines and maintained a normal karyotype after continuous expansion (Figure 1B). All cell lines highly expressed alkaline phosphatase (Figure 1C) and hESC makers Nanog, OCT3/4, SSEA4 and TRA 1-60 ( Additional file 1: Figure S1A). *In vitro* assessment of pluripotency was performed using an embryoid body assay whereby the iPSCs were cultured in suspension under conditions that favored spontaneous differentiation. The cell lines examined were capable of differentiating into cells of mesodermal, endodermal and ectodermal origin ( Additional file 1: Figure S1B), confirming *in vitro* pluripotency. To further evaluate the pluripotency of the iPSC lines, we utilized a functional *in vivo* teratoma formation assay. All of the iPSC lines exhibited the capacity to form tumors in immunocompromised mice that were consistent with teratomas. Tissues representative of the three major germ layers were detected in each tumor (Figure 1D). In addition, we also confirmed the expression of both wild-type and mutant HTT protein in the HD-daughter fibroblasts and derived iPSCs with Western blot analysis ( Additional file 1: Figure S1C).

### HD iPSC-derived neural progenitors form functional neurons *in vitro*

**Figure 2:**
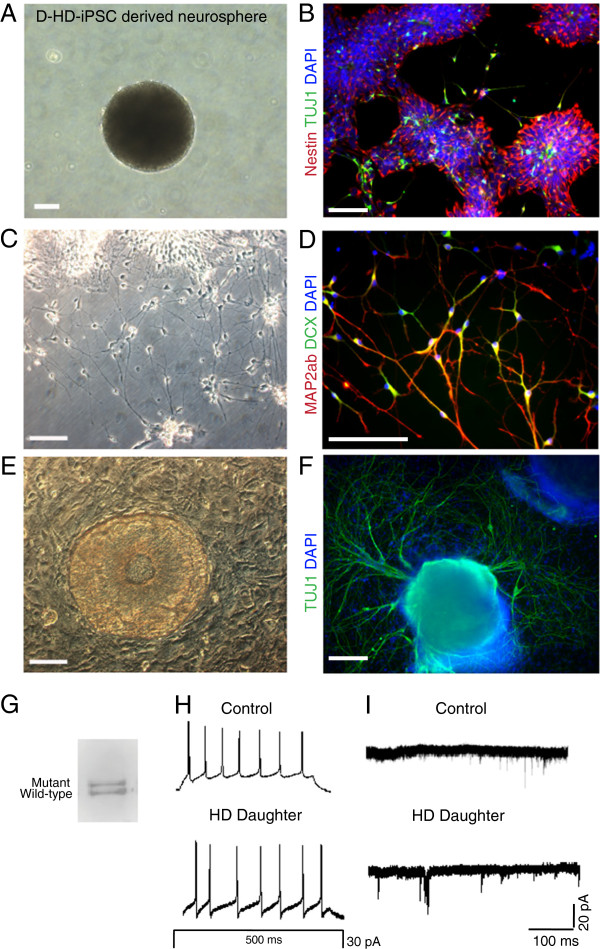
**Differentiation of D-HD-iPSCs into neural progenitors, immature and mature functional neurons. A**: A sample image of a neurosphere generated from the D-HD-iPSC line using the feeder-free differentiation protocol. **B**: Nestin-positive neural progenitors displaying rosette formation and immature (TUJ1^+^) neurons. **C**: Phase contrast image of neurons derived from D-HD-iPSCs. **D**: Mature (MAP2ab^+^) and doublecortin (DCX^+^) expressing neurons. **E**: Neural progenitor derived from D-HD-iPSCs using the PA6 cell line. Stromal cell monolayer observed in the background. **F**: Large networks of TUJ1^+^ neurons emanating from neural progenitors using the PA6 stromal cell line for induction. Scale bars: 100 μm. **G**: D-HD-iPSC derived neurons express both mutant and wild type HTT proteins by Western blot analysis. **H**: Action potentials recorded from neurons derived from D-HD-iPSCs and from C1-iPSCs. **I**: Miniature excitatory post-synaptic currents (mEPSCs) recorded from iPSC-derived neurons.

To assess the neurodevelopmental potential of the HD-iPSC lines, we employed two approaches to differentiate iPSCs into neurons: a feeder-free method using an iPSC-aggregate intermediate to form neural progenitors [36] and a co-culture protocol that uses a mouse stromal cell line to induce neural differentiation [37]. For the feeder-free method, iPSC colonies were grown in suspension in the presence of neural induction medium to generate neurospheres that could be maintained and further passaged (Figure 2A). When plated on poly-l-ornithine-laminin (PLO) coated plates, neurospheres attached and formed neural rosettes expressing neural progenitor marker nestin (Figure 2B). The neural rosettes gave rise to neurons that expressed the neuronal marker beta III tubulin (TUJ1; Figure 2B). For terminal differentiation, the neural progenitors cells were dissociated into single cell suspension and plated on PLO coated plates or coverslips in the presence of neural differentiation medium. Differentiated cells exhibited a typical neuronal morphology (Figure 2C) and expressed neuronal markers microtubule associated protein 2ab (MAP2ab) and/or doublecortin (DCX; Figure 2D). No significant morphological differences were observed for neurons generated from either F-HD-iPSCs or D-HD-iPSCs under the culture conditions, compared to neurons derived from control C1-iPSCs.

For the co-culture method of neural differentiation, iPSCs were mechanically dissociated into small colonies and seeded onto the mouse stromal cell line PA6, which has been shown to induce neural differentiation of hESCs [37,38]. Within one week of culture on PA6, iPSCs differentiated into colonies with characteristic neural progenitor morphology (Figure 2E). After three weeks of co-culture, many cells exhibited neuronal morphology and expressed TUJ1 (Figure 2F). Both HD- and C1-iPSCs were capable of differentiating into neurons via co-culture with the PA6 stromal cell line. Furthermore, both wild-type and mutant HTT proteins were expressed in neural progenitors and neurons derived from the D-HD-iPSCs by Western blot analysis ( Additional file 1: Figure S1C and Figure 2G).

To assess physiological properties of neurons derived from the HD-iPSCs, we performed electrophysiological analysis. Neural progenitor cells derived from each cell line were dissociated into single-cell suspension and plated on a monolayer of rat hippocampal astrocytes for three weeks [39]. Whole-cell patch-clamp recordings showed that neurons from HD-iPSCs exhibited evoked action potentials; similar to those elicited by C1-iPSCs-derived neurons (Figure 2H). Miniature excitatory post-synaptic currents were also detected for HD-iPSC-derived neurons, providing evidence of functional synaptic transmission (Figure 2I). We did not detect overt neuronal functional defects or alterations under the same experimental conditions from either HD-iPSCs.

### HD-iPSC-derived neural progenitors engraft, survive and generate neurons *in vivo*

**Figure 3:**
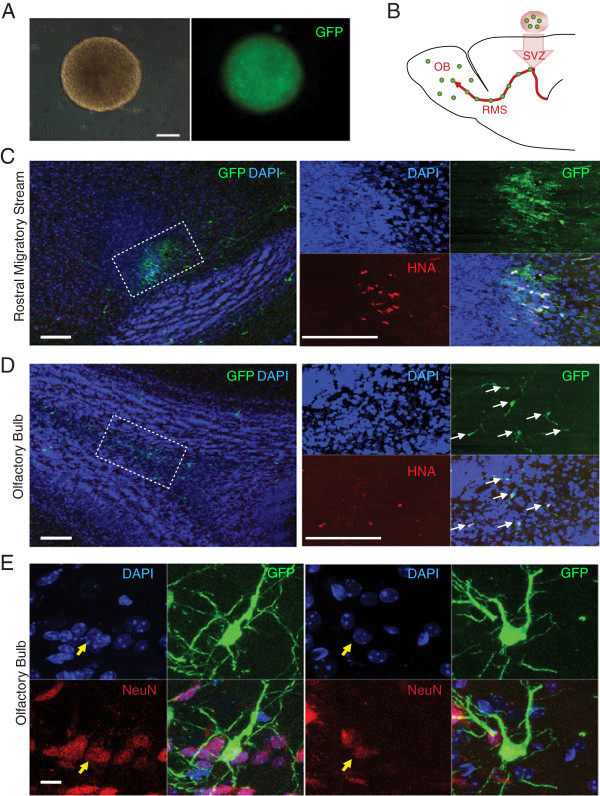
**Transplantation and engraftment of neural progenitors derived from F-HD-iPSCs into the adult mouse brain. A**: Neurospheres derived from F-HD-iPSCs expressing GFP prior to transplantation (phase contrast and fluorescence demonstrating GFP expression). **B**: A diagram illustrating neural progenitor cell transplantation. GFP-labeled neural progenitor cells were injected into the subventricular zone (SVZ) and migrated along the rostral migratory stream (RMS) to the olfactory bulb (OB). GFP and human nuclei antibody (HNA) positive neurons were detected in the RMS (**C**) at six weeks and in the OB (**D**) at eight weeks post-transplantation. **E**: NeuN^+^ GFP expressing cells exhibiting granule cell morphology were detected in the OB eight weeks post-transplantation. Unless otherwise indicated, scale bars: 100 μm.

We next investigated the short-term *in vivo* engraftment potential of HD-iPSC-derived neural progenitor cells by transplanting them into the adult mouse brain. To allow for identification of transplanted cells, we expressed green fluorescent protein (GFP) in F-HD-iPSCs and differentiated them into neural progenitors via the feeder-free method (Figure 3A). Neural progenitor cells were stereotaxically injected into the dorsolateral area of subventricular zone (SVZ) of the lateral ventricles of adult immunecompromised mice (Figure 3B). Adult SVZ provides a neurogenic microenvironment and newly generated neurons from endogenous neural progenitors at SVZ migrate towards the olfactory bulb along a route termed the rostral migratory stream (RMS) [40]. At six to eight week post-transplantation, GFP^+^ cells were found in the RMS (Figure 3C) and the olfactory bulb (Figure 3D) and were positive for human nuclei antibody (HNA), confirming that GFP^+^ cells were originated from transplanted human neural progenitor cells. In addition, some GFP^+^ cells were positive for the neuron-specific nuclear marker NeuN in the olfactory bulb and exhibited morphology typical of mature granule cells (Figure 3E). Thus, neural progenitors derived from HD-iPSCs survive and are capable of differentiating into neurons within the adult mouse brain *in vivo*.

### Astrocytes derived from HD-iPSCs exhibit increased cytoplasmic vacuolation

**Figure 4:**
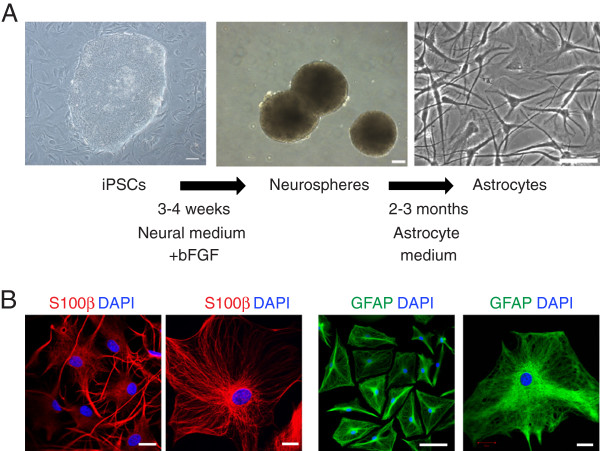
**Generation of astrocytes from iPSCs. A**: A multi-step protocol was used to generate astrocytes. iPSCs were initially differentiated into neurospheres using a feeder-free protocol and subsequently differentiated into astrocytes. **B**: Astrocytes generated from HD-iPSCs exhibited a typical astrocyte morphology and expressed astrocyte markers glial fibrillary acidic protein (GFAP) and S100β. Scale bars: 100 μm (A) or 20 μm (B).

Astrocytes have been shown to exhibit non-cell-autonomous effects on neurons when expressing a HTT protein with a polyglutamine expansion [22,23]. To examine the driect effect of mutant HTT on astrocytes, we differentiated neural progenitors into astrocytes by culturing in the astrocyte medium (Figure 4A). Cells were continually passaged and maintained in the astrocyte medium until they exhibited typical astrocyte morphology. All three iPSC lines were capable of differentiating with similar efficiency into cells that expressed the astrocyte markers glial fibrillary acidic protein (GFAP) and S100β (Figure 4B).

**Figure 5:**
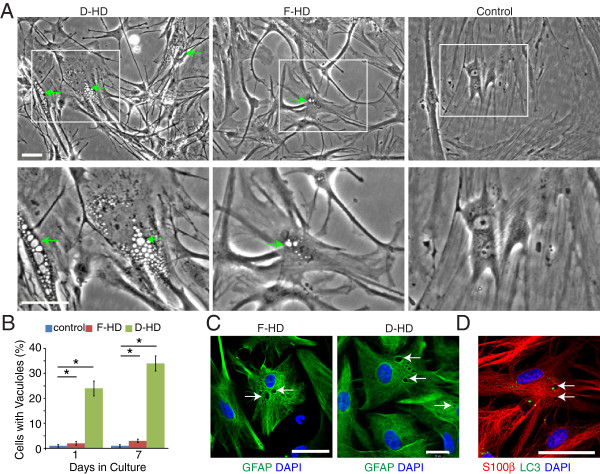
**Astrocytes generated from the D-HD-iPSCs with increased cytoplasmic vacuolation. A**: Vacuoles were detected in the cytoplasm of astrocytes (arrows) under phase contrast microscopy. The number of vacuoles varied depending on the iPSC line used to generate the astrocytes. **B**: Quantification of cytoplasmic vacuolation. Values represent mean + SEM (n = 4; *: *P* < 0.0001; ANOVA). **C**: Cytoplasmic vacuoles appeared empty and were negative for GFAP staining. **D**: The autophagosomal marker LC3 was weakly detected in astrocytes grown under normal culture conditions and rarely colocalized with cytoplasmic vacuoles. Vacuoles often appeared empty (arrows). Scale bars: 50 μm.

In contrast to the absence of an obvious cellular phenotype of neurons with a *HTT* mutation, we detected a rapid accumulation of discrete clear vacuoles of variable number and size within the cytoplasm of astrocytes derived from D-HD-iPSCs (Figure 5A). Similar vacuoles were observed in lower numbers in astrocytes derived from the F-HD-iPSCs. However, vacuoles were rarely observed in C1-iPSC derived astrocytes (Figure 5A). Next, we quantified the percentage of astrocytes that contained cytoplasmic vacuoles. One day after plating, 24% of astrocytes derived from the D-HD-iPSCs already contained cytoplasmic vacuoles, compared to 2.7% of astrocytes derived from the F-HD-iPSCs and 1.1% of control astrocytes (600 cells per line; 4 different experiments). After seven days, the numbers of D-HD astrocytes with cytoplasmic vacuoles increased to 34%, compared to 2.7% of HD-father astrocytes and 1.1% of astrocytes derived from control iPSCs (Figure 5B; 600 cells per line; 4 different experiments).

**Figure 6:**
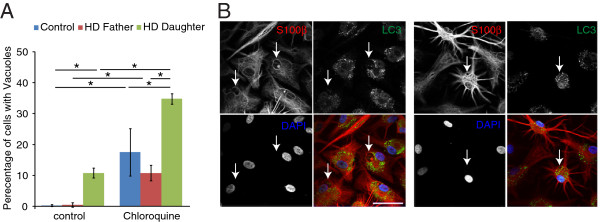
**Enhanced vacuoles formation in astrocytes after chloroquine treatment. A**: Overnight treatment of astrocytes with the autophagy inhibitor chloroquine resulted in increased cytoplasmic vacuolation for all cell lines examined, with D-HD-astrocytes exhibiting the largest number of vacuoles. **B**: S100β expressing astrocytes exhibited increased cytoplasmic staining with the autophagic vacuole marker LC3, after treatment with chloroquine. Scale bar: 50 μm.

The cytoplasmic vacuoles were negative for the intermediate filament protein GFAP and appeared empty (Figure 5C). To determine whether the cytoplasmic vacuoles were autophagosomes, we performed immunocytochemistry for the marker Light Chain 3 (LC3) that binds to autophagocytic membranes [41]. Under basal culture conditions, LC3 staining was weakly detected within the cytoplasm of astrocytes, but rarely colocalize with cytoplasmic vacuoles (Figure 5D). To enhance the detection of autophagosomes, we treated astrocytes overnight with chloroquine, a drug that accumulates in lysosomes and prevents fusion of lysosomes with autophagosome by altering lysosomal pH. Chloroquine treatment significantly increased the number of cytoplasmic vacuoles for all three cell lines examined, with D-HD astrocytes exhibiting the most vacuolation (Figure 6A). The chloroquine-induced vacuoles were mostly autophagsomes that were positive for LC3. However, LC3 did not colocalize with all of the cytoplasmic vacuoles observed (Figure 6B), suggesting the existence of a different type of vacuoles that might associate with the *HTT* mutation in astrocytes.

**Figure 7:**
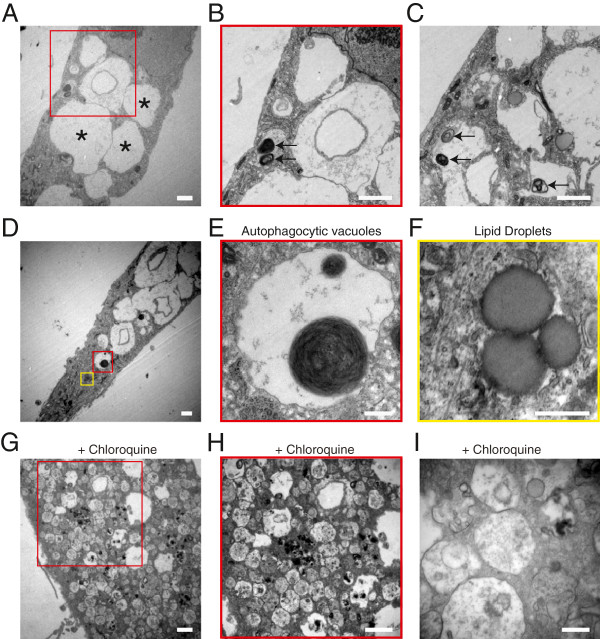
**Ultrastructural changes in astrocytes derived from D-HD-iPSC. A**: Numerous electron clear vacuoles were observed within the cytoplasm of astrocytes. **B, C**: A few autophagocytic vacuoles with myelin whorls (arrows) in astrocytes. **D**: Sample image of an astrocyte with many empty cytoplasmic vacuoles. Higher magnification revealed an autophagocytic vacuole with myelin whorls (**E**) and large spherical cytoplasmic granules representing lipid droplets (**F**). **G, H, I**: Chloroquine treated astrocytes exhibited numerous cytoplasmic vacuolation and proliferation of lysosomes. Scale bars: 2 μm (A-D, G, H) or 500 nm (E, F, I).

To further characterize the cytoplasmic vacuolation observed in HD-astrocytes, we performed transmission electron microscopy (TEM). Ultrastructural examination of astrocytes confirmed the presence of numerous electron clear vacuoles with variable size and shape (Figure 7A). Autophagic vacuoles containing 1-2 myelin whorls were also detected within the cytoplasm of cells, but with much lower abundance (Figures 7B and 7C). Occasionally, we also observed astrocytes with clear empty vacuoles (Figure 7D), autophagosomes (Figure 7E) and dense granules that were compatible with lipid droplets (Figure 7F) coexisting. Consistently, treatment with chloroquine increased the numbers of vacuoles detected within the cytoplasm of astrocytes and many had the appearance of lysosomes that were not electron clear (Figures 7G, 7H, and 7I). Together, these studies suggest that astrocytes with an *HTT* mutation exhibit a rapid accumulation of electron clear vacuoles in a CAG-dose-dependent manner.

## Discussion

HD is a devastating neurological disorder for which there is currently no cure and few therapeutic options. Given the severity of HD, there is a great need for model systems that can be used to further our understanding of the pathogenesis of this disease and as screening tools to discover or evaluate therapeutic compounds. In this study, we developed an *in vitro* model system derived from cells harvested from HD patients with unique genetic relationship between iPSC lines. One iPSC line was derived from a male carrying *an HTT* mutation with 50 CAG repeats and with adult onset HD. The second line was derived from his daughter with an *HTT* mutation of 109 CAG repeats and with juvenile onset HD. Trinucleotide repeat length frequently increases in the offspring of affected fathers, a phenomenon that is accentuated with longer paternal repeat lengths and that accounts for genetic anticipation [15,16]. The underlying mechanism for this genetic instability is not well understood. The HD-iPSCs of a father and child pairs, including cells from the father and the daughter reported here, could potentially be used as a model system to examine this intriguing phenomenon.

The HD-iPSC lines derived in this study exhibit typical hESC morphology, express pluripotency markers, maintain a normal karyotype and generate teratomas *in vivo*[42]*.* All lines were able to form neural progenitor cells, which in turn terminally differentiated *in vitro* into phenotypically normal, functional neurons that could fire action potentials and exhibit functional synaptic transmission. Although mutant HTT protein was present in HD-iPSC-derived neurons, an abnormal neuronal phenotype was not detected under basal conditions [43]. The lack of an obvious abnormality at the neuronal level under normal conditions has been previously reported by investigators utilizing HD-iPSCs [44]. Moreover, a general lack of neuronal phenotype has indicated in studies using iPSCs derived from patients with other neurodegenerative diseases, such as dopaminergic neurons for Parkinson’s disease (PD) [45] and motor neurons for ALS [46]. These findings suggest that simply directing iPSCs to a specific neuronal subtype may not be sufficient to produce disease phenotype for certain aging-dependent neurological disorders and that extensive environmental manipulation or extended observation in culture may be required. For instance, cellular stressors leads to toxicity of HD iPSC-derived neurons under medium spiny neuron differentiation protocol [43]. The environmental cues needed to induce neurodegeneration in HD patients may be complex, requiring an intricate milieu of growth factors, morphogens and appropriate stressors. It may be necessary to further define the *in vitro* culture conditions to fully recapitulate the neuronal pathology observed in patients, such as the formation of HTT cellular inclusions.

The inability to maintain human neurons for long period of time has limited our ability to model late onset neurological diseases in culture; however, the development of patient specific iPSC technology has opened up the possibility of combining animal model systems with cell transplantation for longitudinal studies. We assessed the short-term *in vivo* engraftment capability of F-HD-iPSCs. We demonstrated that neural progenitors derived from F-HD-iPSCs transplanted into neurogenic SVZ of adult NOD-SCID mice were able to migrate along the RMS and generate neurons in the olfactory bulb. Importantly, the HD-iPSC derived cells were able to survive in the mouse brain for at least eight weeks post-transplantation. The results of our xenotransplantation experiments provide a foundation for future long-term engraftment studies and such a system could potentially be utilized to evaluate long term neuronal survival and degeneration *in vivo* and as a preclinical *in vivo* model for testing of therapeutic treatments.

Astrocytes are an abundant cell type in the adult brain and they perform essential and complex functions such as providing metabolic and trophic support for neurons, as well as overall structural support [47,48]. Astrocyte dysfunction has been implicated in the pathogenesis of several neurodegenerative diseases, including ALS [49,50], PD [51] and Alzheimer’s disease [52]. The contribution of astrocytes to the development and progression of HD remains to be elucidated [9,22]. Recent studies have shown that expression of mutant N-terminal Huntingtin fragment in astrocytes suppresses BDNF secretion [53]. One of the advantages of using patient-derived iPSCs to develop an *in vitro* experimental system is the ability of the iPSC to generate any cell types of interest [31]. We utilized the pluripotent property of iPSCs and differentiated them into GFAP^+^ and S100β^+^ astrocytes. Strikingly, discrete, variably sized, clear vacuoles were detected within the cytoplasm of astrocytes derived from both HD-iPSCs, with D-HD-astrocytes had significantly higher number of cytoplasmic vacuoles. When examined by electron microscopy, the cytoplasm contained clear vacuoles that appeared empty, in addition to lower numbers of autophagic vacuoles and lipid droplets. Interestingly, cytoplasmic vacuolation has been observed in primary lymphoblasts harvested from individuals with HD and a correlation was found between the CAG repeat-length and the number of cytoplasmic vacuoles [54,55]. Consistent to what we observed in astrocytes, ultrastructural findings of the lymphoblasts were found to exhibit increased numbers of empty vesicles, autophagocytic vacuoles and lipid droplets.

Successful modeling of neurological disorders using iPSC technology have been reported, but mainly with neurodevelopmental disorders, such as spinal muscular atrophy [56], Rett syndrome [57] and familial dysautonomia [58]. Although many disease-specific iPSC lines for neurodegenerative diseases have been generated, few published studies have identified a disease-related phenotype [31]. In our study, we identified a phenotype in astrocytes generated from iPSCs derived from patients with the neurodegenerative disorder HD. Notably, this phenotype was detected under basal conditions without additional stressors. Importantly, the characteristics of vacuoles in astrocytes was consistent with those seen in primary lymphoblasts harvested from HD patients [54,55], suggesting cellular vacuolation may be a disease associated abnormality that may be used as a biomarker for HD. Future studies will focus on further determining the disease-relevance and mechanistic studies to explore the relationship of the phenotype to HD pathogenesis.

## Conclusion

We have derived patient specific iPSCs from a daughter and her father affected with HD, which provide a unique *in vitro* system to study HD, a defined, genetic, neurodegenerative disorder. The iPSCs from both diseased cell lines were differentiated into neural progenitor cells and further into functional neurons *in vitro,* and give rise to neurons in the adult mouse brain after transplantation *in vivo*. Astrocytes derived from HD iPSCs exhibit increased cytoplasmic vacuolation. Most importantly, we were able to recapitulate a phenotype under basal conditions that has previously been documented in primary cells harvested from HD patients. These results pave the way for future mechanistic studies of HD pathogenesis, disease modeling and for high-throughput therapeutic screens.

## Methods

### Induced pluripotent stem cell derivation and characterization

Skin biopsies were obtained from a male patient with adult onset Huntington’s disease (50 CAG repeats) and his daughter with juvenile onset HD (109 CAG repeats). Dermal fibroblast cultures were established from skin biopsies for iPSC derivation as previously described [59]. Unrelated neonatal foreskin fibroblasts were acquired from American Type Culture Collection (CRL-2097, 28 CAG repeats) for generation of control iPSCs. iPSC lines were derived by retroviral transduction of the four factors (Oct3/4, Sox2, c-MYC and Klf4) according to previously published methods [60]. The iPSC lines were maintained on irradiated mouse embryonic fibroblasts (MEF) in hESC medium (hESM) consisting of Dulbecco’s modified Eagle medium (DMEM)/F12 (Invitrogen) supplemented with 20% Knock-out serum replacement (KOSR, Invitrogen), 2 mM L-glutamine, 0.1 mM β-mercaptoethanol, 0.1 mM non-essential amino acids solution (NEAA), and 8 ng/ml of recombinant human basic fibroblast growth factor (bFGF, Preprotech). G-banding for karyotype analysis was performed for all iPSC lines by the Cytogenetics Core of the Johns Hopkins Medical Institution. All procedures followed approved protocol by Institutional IRB and ISCRO Committees.

Pluripotency marker expression was assessed with immunohistology as previously described [59,61]. Cells were fixed with 4% paraformaldehyde for ten minutes at room temperature, washed with phosphate buffered saline (PBS) and blocked with 10% donkey serum in 0.25% Triton X-100 in PBS for one hour. Specimens were incubated in a solution of 1% donkey serum in PBS overnight at 4°C with the following primary antibodies: goat anti-Nanog (R&D Systems, 1:250), mouse anti-TRA 1-60 (Millipore, 1:250), mouse anti-SSEA4 (Millipore, 1:400), mouse anti-OCT3/4 (Santa Cruz, 1:400). Following three washes with PBS, the samples were incubated with the appropriate secondary antibody in PBS with 1% donkey serum for one hour at RT. Samples were washed with PBS and counter-stained with 4′,6-diamidino-2-phenylindole, dihydrochloride (DAPI) to visualize the nuclei. FITC- and Cy3-conjugated secondary antibodies were obtained from Jackson ImmunoResearch. Images were acquired with confocal microscopy system. Alkaline phosphatase activity was detected using SigmaFAST BCIP/NBT kit (Sigma) according to the manufacturer instructions.

For *in vitro* assessment of pluripotency, an embryoid body assay was performed as previously described [59]. iPSCs were enzymatically detached from MEF by incubation with collagenase IV (Invitrogen). The colonies were washed with PBS (Invitrogen), re-suspended and cultured in suspension using ultra-low attachment plates (Corning). After two days, the medium was changed to DMEM/F12 supplemented with 20% fetal bovine serum (FBS, HyClone) 2 mM L-glutamine, 0.1 mM β-mercaptoethanol, 0.1 mM NEAA. Two weeks after the initiation of embryoid body formation, the aggregates were cultured on gelatin coated plates to allow for attachment, outgrowth and further differentiation. Primary antibodies used for germ-layer evaluation included: mouse anti-human α-fetoprotein (Millipore, 1:250), rabbit anti-nestin (Millipore,1:250) and mouse anti-human α-smooth muscle actin (Millipore,1:500).

To assess the *in vivo* pluripotency of iPSC lines generated, teratoma formation assays were performed as previously described [62]. Four to six week old female NOD.CB17-*Prkdc*^*scid*^*/*J mice (Jackson Laboratory) were injected subcutaneously into the dorsal flank with cells collected from one confluent six-well plate. Cells were harvested by incubation with collagenase IV (Sigma) and resuspended in DMEM/F12 medium supplemented with 30% Matrigel (BD Biosciences). Animals were monitored frequently and visible tumors were excised between eight to 12 weeks post-injection. The tissues were fixed with 10% neutral buffered formalin, paraffin embedded, sectioned and stained with hematoxylin and eosin for histological evaluation. All animal procedures were approved by the Animal Care and Use Committee at Johns Hopkins University School of Medicine.

### Assessment of HTT expression

For determination of HTT expression, cell lysates were prepared in RIPA buffer (1%NP-40, 0.1% SDS, 0.5% sodium deoxycholate in PBS) with protease inhibitor cocktail tablets (Roche). Protein concentrations were determined using the Pierce BCA protein assay kit (Thermo Scientific).To ensure separation of the wild-type and mutant HTT proteins, precast 3-8%Tris-Acetate gels (Invitrogen) were run at 70V for 30 minutes followed by 110V for 9 hours at 4°C. Western blot analysis was performed using mouse anti-HTT antibody (Millipore MAB 2166, 1:10000) and donkey anti-mouse HRP conjugated secondary antibodies (Jackson ImmunoResearch, 1:5000).

### Neural differentiation of iPSCs and assessment of functionality

Neural differentiation of iPSCs was performed using two methods: a feeder-free and a feeder-dependent method. We used a modified version of a previously described feeder-free method [36]. Briefly, iPSCs were enzymatically detached from MEF by incubation with collagenase IV. The colonies were washed with PBS, re-suspended in hESM supplemented with 20 ng/ml of bFGF and cultured in suspension using ultra-low attachment plates. After five days, the medium was changed to a neural-induction medium consisting of neurobasal medium (Invitrogen) supplemented with 2% B27 (Invitrogen), 1% glutamine, and 40 ng/ml of bFGF. Partial media change was performed every second day and colonies were observed for a neurosphere morphology. For terminal differentiation into neurons, neural progenitor cells were mechanically dissociated by trituration to a single cell suspension and plated on poly-L-ornithine (Sigma, 20 μg/ml) and laminin (BD Biosciences, 10 μg/ml) coated coverslips in 24-well plates at a concentration of 30,000 cells/well. To initiate differentiation, dissociated neural progenitor cells were cultured in neural induction medium without bFGF (neural differentiation medium). Culture medium was changed every three to five days and cells were monitored frequently for neuronal growth. In the absence of bFGF, neuronal differentiation was observed at one week post-plating. To confirm neural differentiation, following primary antibodies were used: mouse anti-Nestin (Chemicon, 1:250), rabbit anti-β-tubulin (TUJ1; Sigma 1:2000), mouse anti-MAP2ab (Sigma, 1:250) and goat anti-DCX (Santa Cruz Biotechnology, 1:500).

For neural differentiation via the feeder-dependent method, the PA6 stromal cell line (RIKEN) was utilized according to a previously published protocol [37]. PA6 cells were maintained in Minimum essential medium alpha medium (Invitrogen) supplemented with 10% FBS and 1% penicillin-streptomycin (P/S). Once the stromal cell line had formed a confluent monolayer, it was used for neural differentiation. iPSCs were mechanically harvested from MEFs using the STEMPRO EZPassage tool (Invitrogen), washed with PBS and resuspended in neural induction medium consisting of Glasgow minimum essential medium (Invitrogen), 10% KOSR, 2 mM L-glutamine, 1 mM sodium pyruvate and 0.1 mM β-mercaptoethanol and 1% P/S. Small iPSCs colonies were seeded onto the PA6 monolayer and medium was changed every three days. The cultures were monitored daily for neuronal differentiation.

To assess neuronal function, electrophysiology of neurons derived from control iPSCs and HD-derived iPSCs was performed as previously described [39]. Neural progenitors derived by the feeder-free method were dissociated by trituration and single cells plated onto a layer of rat hippocampal astrocytes [47,63,64]. For whole-cell patch-clamp recordings, the patch pipette (3–7 MΩ) was filled with the following internal solution to record action potentials and mEPSCs: (in mM) K-gluconate 130, KCl 4, HEPES 10, EGTA 2, ATP 4, GTP 0.3, and phosphocreatine 7 (pH 7.3) and the external solution had the following composition (in mM): NaCl 140, KCl 3, CaCl_2_ 2, MgCl_2_ 1.3, HEPES 10, and glucose 10 (pH 7.4). For mEPSC recordings, 10 μM bicuculline and 1 μM tetrodotoxin was added to the external solution. An Axopatch 200B amplifier (Axon Instruments/Molecular Devices Corp., Union City, CA) was used for neuron recordings and analyzed using Clampfit 9.02 (Axon Instruments). Data were digitized at 10 kHz with a 2 kHz low-pass filter.

### Transplantation of iPSC-derived neural progenitors and assessment of engraftment

GFP^+^ neurospheres derived from the F-HD-iPSC line were mechanically dissociated into a single-cell suspension by trituration with a pipette tip. Cells were pelleted by centrifugation and re-suspended in Neurobasal medium at a concentration of 100,000 cells/μl. Four to six week-old female NOD.CB17-*Prkdc*^*scid*^*/*J mice were used as transplantation recipients. Mice were anesthetized and 400,000 cells were stereotaxically injected into the subventricular zone of each mouse (4 μl/site; AP: 1 mm, ML: 1 mm, DV: 2 mm from Bregma). Mice were sacrificed at three, six and eight weeks of age to assess engraftment of the neural progenitor cells. Brain sections were prepared from transplanted mice and processed for immunostaining as described [65,66]. The following primary antibodies were used for immunohistochemistry: mouse anti-NeuN (Chemicon, 1:500), rabbit anti-GFP (1:1000) and mouse anti-human nuclei (HNA,Millipore, 1:100). Images were acquired on a META multiphoton confocal system (Zeiss LSM 510) using a multi-track configuration. Procedures were performed in accordance with the Animal Care and Use Committee at Johns Hopkins University.

### Astrocyte differentiation of iPSCs and characterization

Neurospheres derived using the feeder-free method were mechanically dissociated into a single cell suspension, filtered through a cell strainer and plated on poly-L-ornithine-laminin coated flasks. Cells were cultured in Astrocyte medium (ScienCell) and passaged as necessary. Once cells exhibited astrocyte morphology and formed a continuous monolayer, they were evaluated for the expression of astrocyte markers by immunocytochemistry using rabbit anti-GFAP (Dako, 1:500) and mouse anti-s100β (Sigma, 1:500). To quantify the number of astrocytes with cytoplasmic vacuolation, 600 randomly chosen cells were examined for each cell line and the average percentage of cells with vacuoles for four experiments was determined. Astrocytes were also examined for the presence of autophagosomes by immunostaining for the autophagy marker LC3 using rabbit anti-LC3 A/B (Cell Signaling, 1: 200). To induce autophagosome formation, astrocytes were treated with 50 μM chloroquine diphosphate (Invitrogen) overnight.

### Transmission electron microscopy (TEM)

Cells were fixed in 2.5% glutaraldehyde, 3mM CaCl2, 1% sucrose, in 0.1 M sodium cacodylate buffer (pH 7.2) for one hour at RT. After a buffer rinse, samples were post-fixed in 1% osmium tetroxide in buffer (1 hour) on ice in the dark. Specimens were stained with 2% aqueous uranylacetate, dehydrated in a graded series of ethanol and embedded in Eponate 12 (Ted Pella) resin. Samples were polymerized at 60°C overnight. Ultrathin sections were prepared with a diamond knife on the Reichert-Jung Ultracut E ultramicrotome and picked up with naked 200 mesh copper grids. Grids were stained with 2% uranyl acetate in 50% methanol and cells were observed with a Hitachi 7600 TEM at 80 kV. Images were captured with an AMT CCD (1K x 1K) camera. TEM was performed at the Johns Hopkins Institute for Basic Biomedical Sciences microscope facility.

## Competing interests

The authors declare that they have no competing interests.

## Authors’ contributions

T.A.J., R.L.M, C.A.R., G.L.M. and H.S. conceived the study. T.A.J. led the project and performed the majority of experiments. W.R.K. performed the transplantation experiment. C.C. derived iPSC lines. H.Y. performed the electrophysiology analysis. R.L.M. and C.A.R. provided HD patient skin biopsy samples. T.A.J., G.L.M. and H.S. wrote the paper. All authors read and approve the manuscript.

## Supplementary Material

Additional file 1 **Figure S1. *In vitro *characterization of the D-HD-iPSCs. A**: Expression of pluripotency markers. Immunostaining for the nuclear markers nanog and octamer-binding transcription factor 3/4 (OCT3/4) and the cell surface markers TRA 1-60 and stage specific embryonic antigen 4 (SSEA4) were positive. **B**: *In vitro *pluripotency was confirmed using an embryoid body assay to generate cells derived from the three major germ layers. Alpha-fetoprotein (AFP) positive cells representing endoderm, smooth muscle actin (SMA) expressing cells representing mesoderm and nestin positive cells representing ectoderm were identified. Scale bars: 100 μm. **C**: Western blot analysis confirmed the expression of mutant and wild-type HTT proteins in fibroblasts, iPSCs and neurospheres derived from the D-HD-iPSC. The hESC line H1 only expresses one band, corresponding to the wild-type protein. Click here for file
