# Supplementary figures and images for: Astrocytes generated from patient induced pluripotent stem cells recapitulate features of Huntington’s disease patient cells
Source: Mol Brain. 2012 May 21;5:17. doi: 10.1186/1756-6606-5-17 (PMC3506453; doi:10.1186/1756-6606-5-17)

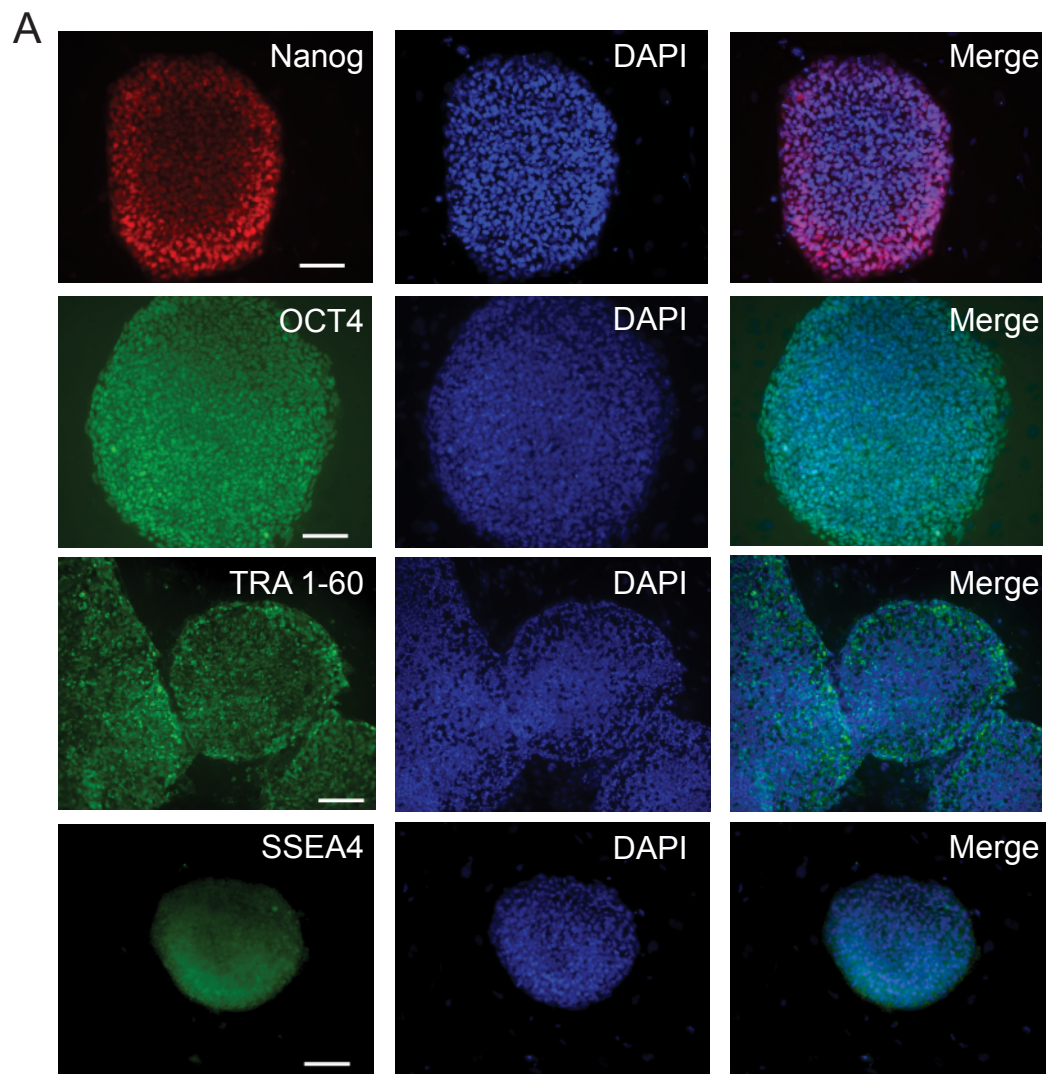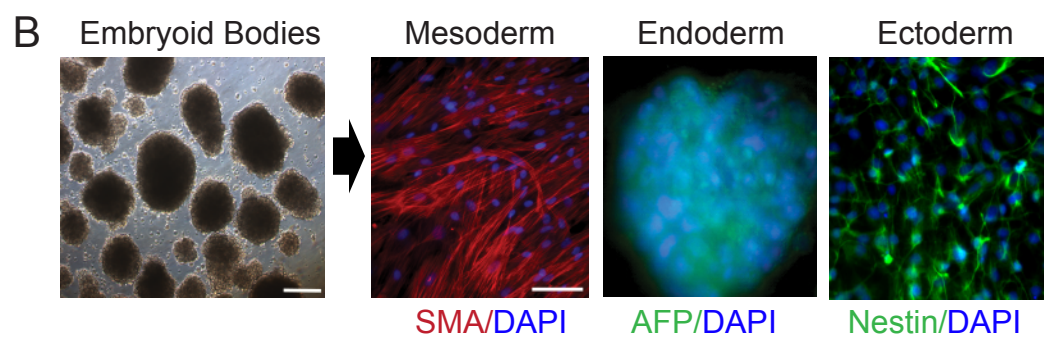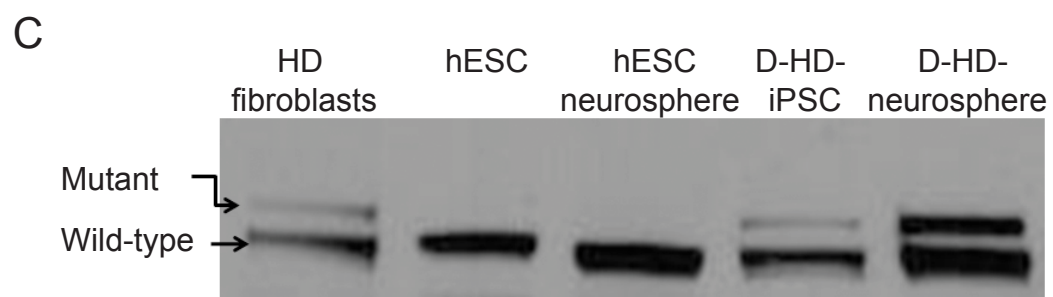

Figure S1 (Juopperi et al.)

Supplement: Additional file 1 — Figure S1. In vitro characterization of the D-HD-iPSCs. A: Expression of pluripotency markers. Immunostaining for the nuclear markers nanog and octamer-binding transcription factor 3/4 (OCT3/4) and the cell surface markers TRA 1-60 and stage specific embryonic antigen 4 (SSEA4) were positive. B: In vitro pluripotency was confirmed using an embryoid body assay to generate cells derived from the three major germ layers. Alpha-fetoprotein (AFP) positive cells representing endoderm, smooth muscle actin (SMA) expressing cells representing mesoderm and nestin positive cells representing ectoderm were identified. Scale bars: 100 μm. C: Western blot analysis confirmed the expression of mutant and wild-type HTT proteins in fibroblasts, iPSCs and neurospheres derived from the D-HD-iPSC. The hESC line H1 only expresses one band, corresponding to the wild-type protein. [file 1756-6606-5-17-S1.pdf]
